# Knowledge of Previous Tasks: Task Similarity Influences Bias in Task Duration Predictions

**DOI:** 10.3389/fpsyg.2018.00760

**Published:** 2018-05-24

**Authors:** Kevin E. Thomas, Cornelius J. König

**Affiliations:** ^1^Department of Psychology, Faculty of Science and Technology, Bournemouth University, Bournemouth, United Kingdom; ^2^Department of Psychology, Faculty of Human and Business Sciences, Saarland University, Saarbrücken, Germany

**Keywords:** task duration prediction bias, planning fallacy, task similarity, previous task duration feedback, selflearning account

## Abstract

Bias in predictions of task duration has been attributed to misremembering previous task duration and using previous task duration as a basis for predictions. This research sought to further examine how previous task information affects prediction bias by manipulating task similarity and assessing the role of previous task duration feedback. Task similarity was examined through participants performing two tasks 1 week apart that were the same or different. Duration feedback was provided to all participants (Experiment 1), its recall was manipulated (Experiment 2), and its provision was manipulated (Experiment 3). In all experiments, task similarity influenced bias on the second task, with predictions being less biased when the first task was the same task. However, duration feedback did not influence bias. The findings highlight the pivotal role of knowledge about previous tasks in task duration prediction and are discussed in relation to the theoretical accounts of task duration prediction bias.

## Introduction

Predicting task duration has been the focus of much research, which has almost universally found that predictions are biased (e.g., Buehler et al., 1997; König et al., 2015). Prediction bias is evident on tasks as diverse as writing a college thesis (Buehler et al., 1994), shopping for gifts (Kruger and Evans, 2004), and solving abstract problems (Thomas et al., 2003). There is considerable support for the planning fallacy, which is the tendency to underestimate future task duration despite knowing that previous similar tasks did not finish on time (Buehler et al., 2010b).

The planning fallacy was identified by Kahneman and Tversky (1979) who suggest that two kinds of data are available when predicting task duration: singular and distributional information. Distributional information is essentially base-rate data (e.g., previous task performance), whereas singular information concerns aspects of a focal task (e.g., number of parts). Kahneman and Tversky (1979) claim that the planning fallacy occurs because singular information becomes the focus of attention whilst distributional information is ignored. This explanation has been termed the inside–outside account (Kahneman and Lovallo, 1993), as using singular or distributional information is like taking an ‘inside’ or ‘outside’ view of a focal task, respectively.

Inherent in the inside–outside account is the notion that people possess distributional information (e.g., they remember their performance on previous similar tasks) but typically ignore it when predicting task duration. Kahneman and Tversky (1979) suggest that when making a prediction on a task that has been encountered before people place less emphasis on what task information they remember and more emphasis on what they perceive differentiates the current task from previous similar tasks. Accordingly, through the use of verbal protocols (Ericsson and Simon, 1980), Buehler et al. (1994) found that predictions are typically based on aspects of focal tasks, but when people are prompted to consider their previous task performance just before making a time prediction, the temporal underestimation indicative of the planning fallacy is reduced. Buehler et al.’s (1994) research suggests that people tend to take an ‘inside’ perspective when making task duration predictions, but when they incorporate what information they remember about previous tasks into their predictions, they do a better job of judging when tasks will finish. Although the inside–outside account makes no prediction about the role memory for previous task performance in the task duration prediction process, the work of Buehler et al. (1994) highlights the benefit of taking such an outside perspective when making time predictions. The role of memory for previous task performance is central to another account of the planning fallacy which claims that people use distributional information but inaccurately recall it when making time predictions (Roy et al., 2005). This memory-bias account states that people consider previous task information but their memory for such information is biased, which leads to biased predictions. Roy and Christenfeld (2007) suggest that because people rarely closely monitor the durations of the tasks they perform in daily life, the planning fallacy (and the overestimation of future task duration) is a result of memory being for estimated or perceived duration rather than actual duration. Support for this claim comes from the retrospective time estimation literature, where autobiographical events (Burt, 1992) and public events (Burt and Kemp, 1991) are remembered as being shorter than was actually the case. There is growing support for the memory-bias account because the amount of prior experience of a focal task seems to matter when it comes to underestimating task duration (Roy and Christenfeld, 2007). Moreover, prediction bias is reduced when feedback about previous task duration is provided, thus correcting memory (Roy et al., 2008). Similar to research supporting the inside–outside account (Buehler et al., 1994), Roy et al.’s (2008) findings suggest that using previous task information can reduce prediction bias, suggesting some complementarity between the memory-bias and inside–outside accounts. However, Roy et al. (2008) found that such information had to be accurate to be beneficial whereas this was not examined by Buehler et al. (1994), suggesting an arguably subtle difference between the two accounts. A more obvious way in which the accounts differ though is in the link between the use of previous task information and prediction bias, with the inside–outside account predicting that bias is due to not using information and the memory-bias account predicting that bias occurs because such information is incorrectly used.

Similar to the memory-bias account, the anchoring account (Thomas et al., 2007), emphasizes the use of information about previous tasks. The account derives from the anchoring and adjustment heuristic (Tversky and Kahneman, 1974) and posits that the actual or perceived duration of previous tasks serves as a basis (anchor) for predictions which are typically insufficiently adjusted according to the demands of focal tasks. This anchoring and insufficient adjustment process results in underestimation when previous tasks are shorter than focal tasks and overestimation when previous tasks are longer than focal tasks. This difference in the direction of bias was found by Thomas et al. (2007) who varied the relative durations of previous and focal tasks. Further support for the anchoring account comes from studies where participants are shown numbers (anchors) representing the durations of previous tasks before predicting the durations of focal tasks. For example, König (2005) found that presenting an anchor concerning a shorter or longer duration on the same task as a focal task resulted in underestimation or overestimation, respectively. Similarly, Thomas and Handley (2008) found that the direction of bias differed in the same way when anchor values concerned a task that was the same as or different to a focal task. Moreover, bias was reduced among participants that reported taking account of their performance on previous similar tasks when making a prediction on the focal task, suggesting that prior task experience might reduce the impact of task duration anchors on prediction bias. However, prior task experience was not manipulated by Thomas and Handley (2008), meaning that the effect of the similarity between anchor value (previous) tasks and focal tasks is unclear.

Anchoring is also posited as a factor in time prediction bias when information about previous task duration is not explicitly presented (e.g., in the form of anchor values). König et al. (2015) suggest that implicitly knowing that one misestimated the duration of a previous task is sufficient to reduce prediction bias on the next task through self-generated feedback on task duration. König et al. (2015) identified this self-learning effect by using two unrelated tasks of similar duration (coloring-in a drawing and building a toy bird) and having some participants estimate the duration of the first task retrospectively. They found that both tasks were underestimated but that the retrospective estimate reduced prediction bias on the second task relative to when no retrospective estimate was made and relative to the first task. König et al. (2015) suggest that making the retrospective estimate led participants to focus attention on how long they thought they took on the first task and they used this information as a self-generated anchor value that was adjusted according to the perceived demands of the second task. Consistent with this claim, self-generated anchor values have been shown to influence the extent of adjustment in judgments on tasks without a temporal element (e.g., general knowledge tests; Epley and Gilovich, 2005), suggesting that an explicit anchor value is not necessary to induce an anchoring and adjustment judgment strategy (Jacowitz and Kahneman, 1995). From their findings, König et al. (2015) proposed the self-learning account of task duration prediction bias.

The self-learning (König et al., 2015), anchoring (e.g., Thomas et al., 2007), memory-bias accounts (e.g., Roy and Christenfeld, 2007), and inside–outside accounts (Kahneman and Lovallo, 1993) all imply that information about previous tasks can influence bias in predictions of task duration. However, unlike the inside–outside account (see e.g., Buehler et al., 1994), the other three accounts suggest that using such information does not necessarily reduce bias. The shared focus on the use of information about previous tasks not necessarily reducing bias implies that the memory-bias, self-learning, and anchoring accounts are complementary.

Although some factors concerning information about previous tasks have been found to influence prediction bias (e.g., temporal distance between actual and predicted duration; Roy and Christenfeld, 2007), a potentially important factor, task similarity, has yet to be studied. The similarity of previous and focal tasks is important and germane to the self-learning, anchoring, and memory-bias accounts because it concerns the relevance of previous task information. Moreover, manipulating task similarity allows the testing of predictions derived from these three accounts plus the inside–outside account. Thus, examining task similarity enhances our understanding of the mechanisms underlying task duration prediction bias and provides an important contribution to the extant literature. Although task similarity should not influence bias according to the inside–outside account (Kahneman and Lovallo, 1993) because information about previous tasks is typically ignored, incorporating remembered information about previous similar tasks into predictions would presumably reduce bias because of the relevance of that information. Thus, a prediction based on the inside–outside account would be that bias is less when tasks are similar, provided that people consider their previous task performance when making predictions (Buehler et al., 1994).

Predicting the effect of task similarity is clearer for the memory-bias account (Roy et al., 2005) because previous task performance is remembered, albeit incorrectly, when making predictions. This process implies that previous similar tasks are relevant to focal tasks and thus informative for predicting task duration, whereas memory for previous different tasks is irrelevant and so of little or no benefit. Furthermore, if people know how long they took on a previous similar task (through receiving feedback), bias should be reduced due to such information correcting memory (Roy et al., 2008). Thus, the memory-bias account would predict that bias is less when tasks are similar but only when the exact task duration of previous tasks is known.

The effect of task similarity can be predicted from the anchoring account because of centrality of the use of previous task duration in the time prediction process to the account. Using previous task duration as an anchor for predictions can occur when previous and focal tasks are different and might or might not reduce bias depending on the relative durations of the tasks (e.g., Thomas et al., 2007). However, such anchoring should only reduce bias when previous and focal tasks are similar because of the relevance of the anchor information (Thomas and Handley, 2008). Thus, the anchoring account would predict that bias is less when previous and focal tasks are similar.

Similarly, because of the centrality of the use of previous task information in the self-learning account, the effect of task similarity can be predicted clearly. Although previous different tasks can influence predictions through learning that such tasks were misestimated and using this knowledge to adapt predictions on a focal task (König et al., 2015), when tasks are similar, this knowledge will be pertinent to the focal task and so reduce bias. Thus, the self-learning account would predict that task similarity reduces bias.

In conjunction with task similarity, examining the role of knowing the exact duration of a previous task through receiving feedback permits further evaluation of the memory-bias, anchoring, and self-learning accounts, all of which emphasize the role of previous tasks in influencing prediction bias. For the memory-bias account, being told how long a previous task took should correct memory, resulting in less prediction bias previous and focal tasks are similar (Roy et al., 2008). For the anchoring account, basing predictions on the feedback exact duration of a previous similar task should reduce bias because of the relevance of that task information to the focal task (Thomas and Handley, 2008). For the self-learning account, thinking about one’s performance on previous task where the exact duration is known should be useful only when the tasks are similar, thus reducing bias due to transferring the insight gained from the previous task to the focal task (König et al., 2015).

The present research comprised three experiments in which student participants performed two tasks in two sessions separated by a temporal interval (1 week) that is reflective of what happens in applied settings (e.g., workplaces). In such settings, there are rarely occasions when tasks are totally novel (i.e., when nothing remotely similar has been done before), with people typically undertaking fairly familiar tasks (Boltz et al., 1998; Hinds, 1999). To try to mimic this state of affairs, the focal task chosen here was one that involved the kind of skills that students would often utilize in the course of their studies (e.g., proofreading and editing assignments). In all experiments, the first and second tasks were the same (formatting an essay twice) or different (building a miniature castle first then formatting an essay second). Prediction bias on the second more familiar task was the measure of the effect of task similarity and previous task duration feedback. Feedback on the exact duration of the first task was provided at the end of the first session in Experiments 1 and 2. In Experiment 2, prompting the recall of that feedback at the start of the second session was manipulated. In Experiment 3, providing feedback on the duration of the first task was manipulated.

All experiments tested the hypothesis that bias on the more familiar second task would be less when the two tasks were identical. Experiment 2 also tested the hypothesis that prompting the recall of the feedback duration of the first task would correct memory, resulting in less biased predictions on the second task when the two tasks identical. Similarly, Experiment 3 tested the hypothesis that having feedback on the exact duration of the first task would correct memory, thereby reducing bias on the second task when the two tasks were identical. Given the shared focus on the use of previous task information not necessarily reducing prediction bias among the memory-bias, self-learning, and anchoring accounts, additional analyses sought to test predictions from these accounts in all experiments.

## Experiment 1

Participants performed either the same essay-formatting task at the first and second sessions or built a miniature castle at the first session and formatted the essay at the second session. Participants were told the exact duration of the task they performed at the first session just after they had finished that task.

### Method

#### Participants

Forty psychology students (28 female and 12 male) at a large university in Southern England participated voluntarily in return for course credit. Participants were aged 18 to 33 (*M* = 20.90, *SD* = 3.46) years.

#### Design

A one-factor [Task Similarity (same vs. different)] between-groups design was used.

#### Materials

A computer-based essay formatting task was chosen as the focal task and the identical previous task because it is typical of the kind of academic task routinely performed by university students and has been used in similar research (Francis-Smythe and Robertson, 1999). The task was a 7-page, 2200-word essay, which was a template from a module on a History degree course. Its text was black, Times New Roman, 12-point font, and had 1.5 line-spacing. Each page was A4-sized with 1-inch margins all around. Formatting the unformatted essay involved making 200 changes ranging from correcting spelling and grammatical errors to changing the typeface (e.g., italicizing text).

A Playmobil^®^ plastic toy was chosen as the different previous task because it has been used in task duration prediction research (Thomas et al., 2007). The task involved constructing a model multi-turreted castle comprising 68 components by following a set of pictorial sequential instructions presented in a booklet.

#### Procedure

Participants were recruited to an experiment on task performance and were not informed of the time estimation element of it until the end of the second session. At the first session, participants were randomly assigned to one of the two equal-sized first task conditions: essay and castle. At each session, participants were tested individually in a laboratory with no clock, and were instructed to remove their watches and place them out of sight so that they would not be a distraction during task performance.

At the first session, before predicting task duration, participants in the castle condition were informed that they could refer to the instruction booklet whilst building the castle. They were then given 2 min to view the instruction booklet and task components that were arranged on a table in front of them. At the first session, the essay condition was presented with a paper copy of the formatted and unformatted versions of the essay task and given 2 min to inspect them before predicting task duration. Afterwards, the paper copy of the unformatted task was removed from sight and a laptop computer with the unformatted task displayed on its screen (as a Microsoft^®^ Word Version 2000 document) was placed on a table in front of these participants. Performing the task involved making the necessary changes to the on-screen unformatted task so that it looked exactly like the paper version of the formatted task that was located beside the computer and had to be followed.

Before performing each task, participants predicted task duration (in whole or part minutes) in writing. Participants were asked to make as accurate and realistic a prediction as possible and were allowed as much time as they needed to do this. The sheets on which predictions were made were removed from sight whilst the task was performed so that predictions could not be amended. To facilitate thorough task performance, after predicting duration, participants were informed that the task would be inspected for accuracy after completion. Task duration was recorded using a digital stopwatch and participants were informed of their completion times just after the task had finished.

This procedure was repeated for the second session, except that all participants performed the essay task from the first session and no feedback was given about its duration. At the second session, participants that performed the castle task at the first session were in the castle-first condition and participants that performed the essay task at the first session were in the essay-first condition. At the end of the second session, participants were fully debriefed about the study. Each session lasted between 40 and 50 min.

### Results and Discussion

The data from Experiment 1 is available in **Supplementary Data Sheet S1**. At the first session, all the castle condition built the castle correctly and the essay condition made an average of 181.75 (*SD* = 8.87) changes to the essay. At the second session, the number of changes to the essay did not differ between the essay-first (*M* = 189.00, *SD* = 4.63) and castle-first conditions (*M* = 186.80, *SD* = 4.95), *t*(38) = 1.45, *p* = 0.155, *d* = 0.46, suggesting that any difference in prediction bias found at the second session is not due to the number of changes made to the essay.

Basic descriptive statistics are presented in **Table 1**, which shows that there was underestimation on the essay and overestimation on the castle at the first session. The tasks differed in duration, with the castle taking just over half as long as the essay, *t*(38) = 5.38, *p* < 0.001, *d* = 1.70. At the second session, **Table 1** shows that there was overestimation in the essay-first condition and underestimation in the castle-first condition. **Table 1** shows that the duration of the second essay task differed between the conditions, with the castle-first condition taking 20 to 25% longer than the essay-first condition, *t*(38) = -2.99, *p* = 0.005, *d* = 0.94.

**Table 1:** Mean (standard deviation) predicted and actual duration and prediction bias (minutes), and proportional error scores per task and task similarity condition in Experiment 1.

Task		Task similarity condition	
		Essay-first	Castle-first
		(*n* = 20)	(*n* = 20)
First	Predicted duration	15.62 (9.43)	20.45 (7.63)
	Actual duration	33.75 (9.86)	18.84 (7.50)
	Prediction bias	-18.13 (13.08)	1.61 (7.59)
	Error score	-0.50 (0.32)	0.16 (0.38)
Second (Essay)	Predicted duration	33.40 (19.91)	19.70 (11.05)
	Actual duration	30.11 (7.85)	38.14 (9.13)
	Prediction bias	3.29 (15.94)	-18.44 (14.33)
	Error score	0.08 (0.40)	-0.46 (0.31)

As actual task durations were not similar between the conditions in both sessions, proportional error scores were computed to assess prediction bias. These scores measure prediction bias as a function of task duration and are calculated by subtracting predicted from actual task duration and dividing the difference by actual task duration. Proportional error scores are well-established as an appropriate dependent measure in the time estimation literature (Brown, 1997).

On the first task, prediction bias was less on the castle, *t*(38) = -5.96, *p* < 0.001, *d* = 1.87. One-sample *t*-tests with values of zero (no bias) revealed significant underestimation on the essay, *t*(19) = -7.04, *p* < 0.001, and non-significant overestimation on the castle, *t*(19) = 1.89, *p* = 0.075. As the essay task involves the kind of skills that students are likely to use when preparing college assignments (e.g., proofreading), significant underestimation on the task could be due to participants recalling how they performed on previous similar tasks and using this knowledge as a basis for their prediction. If such knowledge was inaccurately recalled, then significant underestimation would be expected according to the memory-bias account (see Roy and Christenfeld, 2007). Similarly, a lack of knowledge of the seemingly less familiar castle task should result in less biased predictions because of the lack of task-specific memories to inaccurately recall. On tasks where prior task experience is low or absent, predictions would presumably be based on information concerning the task at hand such as the number of distinct, discrete elements the task entails (Thomas et al., 2003). On the second task, prediction bias was less in the essay-first condition, *t*(38) = 4.97, *p* < 0.001, *d* = 1.51, with significant underestimation in the castle-first condition, *t*(19) = -6.50, *p* < 0.001, and non-significant overestimation in the essay-first condition, *t*(19) = 0.88, *p* = 0.389. The effect of task similarity was further examined by comparing predictions on the second task. Predictions were longer in essay-first condition than the castle-first condition, *t*(38) = 2.69, *p* = 0.011, *d* = 0.85, suggesting that the effect was driven by the difference in predictions and actual durations (see above).

To assess whether predictions were based on previous task duration (Roy et al., 2005), the relationship between predictions on the second task and the actual, feedback duration of the first task was analyzed. Predictions were more highly correlated with actual durations in the essay-first condition, *r*(20) = 0.71, *p* < 0.001, than the castle-first condition, *r*(20) = 0.20, *p* = 0.405, and this difference was significant, Fisher *z* = 1.99*, p* = 0.046. These findings suggest that knowing the exact duration of a previous task is informative for predictions only when this knowledge is relevant to the focal task. In conjunction with the effect of task similarity, these findings indicate that feedback on previous task duration does not debias predictions when it pertains to a different task (Roy et al., 2008).

To assess whether predictions were anchored on previous task duration, the difference between predictions on the second task and actual durations on the first task was analyzed per condition. The reason for this analysis is that anchoring involves using the feedback duration of the first task as a basis for predicting the duration of the second task. Thus, predictions should be close to actual durations if an anchoring strategy is used (Thomas et al., 2007). Predictions were found to be similar to actual durations in the essay-first condition, *t*(38) = 0.11, *p* = 0.915, *d* = 0.03, and the castle-first condition, *t*(38) = -0.32, *p* = 0.754, *d* = 0.07, suggesting that anchoring occurred. Using an anchoring strategy should result in slight overestimation in the essay-first condition because those participants tended to complete the second task faster than the first task. Conversely, in the castle-first condition, such anchoring should result in underestimation on the essay as the essay was longer than the castle. In conjunction with the task similarity effect, these findings provide support for the anchoring account but indicate that anchoring only reduces misestimation when a previous task is the same as a focal task (Thomas and Handley, 2008).

To assess whether learning from prediction bias on the first task and using this self-generated feedback as a basis for predictions on the second task (König et al., 2015) was influenced by task similarity, error scores on the first and second tasks were compared between the task similarity conditions. A 2 (task) × 2 (task similarity) mixed ANOVA produced an interaction, *F*(1,38) = 7.24, *MSE* = 0.12, *p* < 0.001, $\eta_{p}^{2}$ = 0.62, with error scores being higher on the first task than the second task in the essay-first condition (*p* < 0.001) and lower on the first task than the second task in the castle-first condition (*p* < 0.001). In conjunction with the task similarity effect, this finding indicates that participants learnt from their mistakes on the first task when it was the essay and used this self-generated informative feedback to make a less biased prediction when faced with the same task again. Thus, there is support for the self-learning account (König et al., 2015) and the idea that the transfer of insight gained on a previous task is only useful when that task is the same as a focal task.

This experiment highlights the effect of information about previous tasks on task duration prediction bias and provides support for the memory-bias, anchoring, and self-learning accounts. However, the effect of previous task information was examined indirectly through telling all participants how long they took on the first task. Thus, it was not possible to directly assess whether such feedback was used when predicting task duration. To provide a stronger test of the effect of previous task information, the explicit recall of the feedback duration of the first task was manipulated alongside task similarity in Experiment 2.

## Experiment 2

Participants performed two tasks (formatting an essay twice or building a miniature castle then formatting the same essay) 1 week apart. At the end of the first session, the exact duration of the first task was feedback to all participants. At the second session, half of those participants that built the castle and half of those that formatted the essay at the first session were prompted to recall the feedback duration of the first task.

Following Experiment 1, it was hypothesized that: (1) prompting the recall of the duration of the first task would reduce prediction bias on the second task when the two tasks were identical; (2) predictions on the second task would be less biased when the two tasks were identical.

### Method

#### Participants

Eighty psychology students (23 male and 57 female) at a large university in Southern England participated voluntarily in return for course credit. Participants were aged 18 to 36 (*M* = 23.16, *SD* = 4.51) years.

#### Design

A 2 (Task Similarity [same vs. different]) × 2 (Previous Task Duration Memory Prompt [yes vs. no]) between-groups design was used.

#### Materials

The essay task was from Experiment 1. The castle task was a Lego plastic toy that involved constructing a miniature castle tower comprising 164 components. This task comprised more components and so was considered to be more complex. Moreover, pilot-testing (*N* = 8) revealed that the castle task took 32.60 min on average to complete (*SD* = 4.68), meaning that it was of similar duration to the essay task from Experiment 1 and of longer duration than the castle task from Experiment 1.

#### Procedure

The procedure was the same as Experiment 1, except for the following two changes. One, at the start of the first session, participants were randomly assigned to one of four equal-sized conditions that were formed by crossing the previous task duration memory prompt and task similarity factors. Two, at the start of the second session before predicting task duration, the memory prompt conditions were asked to recall the duration of the first task, of which they had been informed at the end of the first session. Half of these participants recalled task duration within 10 min of actual task duration and 20% recalled task duration exactly.

### Results and Discussion

The data from Experiment 2 is available in **Supplementary Data Sheet S2**. At the first session, the essay condition made 159.95 corrections to the task on average (*SD* = 24.39) and all of the castle condition completed the task correctly. At the second session, the number of changes made to the essay task did not differ between the essay-first (*M* = 163.55, *SD* = 19.70) and castle-first conditions (*M* = 164.35, *SD* = 22.63), *t*(78) = -0.17, *p* = 0.867, *d* = 0.04.

Basic descriptive statistics for the first and second tasks are presented in **Tables 2**, **3**, respectively. **Table 2** shows that there was underestimation on the castle and the essay at the first session. The tasks differed in duration, with the essay taking longer than the castle, *t*(78) = 5.52, *p* < 0.001, *d* = 1.24. **Table 3** shows that there was overestimation in the essay-first conditions and underestimation in the castle-first conditions on the second task. The castle-first conditions took longer to complete the essay (*M* = 39.30, *SD* = 11.37) than the essay-first conditions (*M* = 34.55, *SD* = 8.54), *t*(78) = 2.11, *p* = 0.038, *d* = 0.47. Due to the difference in duration of the tasks at sessions 1 and 2, error scores were used to assess prediction bias.

**Table 2:** Mean (standard deviation) predicted and actual duration and prediction bias (minutes), and proportional error scores per the first task in Experiment 2.

	Task	
	Essay (*n* = 40)	Castle (*n* = 40)
Predicted duration	27.63 (13.21)	25.95 (12.43)
Actual duration	42.43 (10.50)	29.80 (9.93)
Prediction bias	-14.80 (17.23)	-3.85 (14.96)
Error score	-0.29 (0.43)	-0.05 (0.53)

**Table 3:** Mean (standard deviation) predicted and actual duration and prediction bias (minutes), and proportional error scores per task similarity by memory prompt condition on the second task in Experiment 2.

	Condition			
	Essay-first No prompt	Essay-first Prompt	Castle-first No prompt	Castle-first Prompt
	(*n* = 20)	(*n* = 20)	(*n* = 20)	(*n* = 20)
Predicted duration	36.05 (9.77)	35.80 (10.58)	30.05 (7.75)	32.40 (7.59)
Actual duration	35.60 (9.33)	34.00 (7.13)	37.75 (9.22)	40.85 (13.25)
Prediction bias	0.45 (6.98)	1.80 (11.04)	-7.70 (10.95)	-8.45 (15.89)
Error score	0.06 (0.30)	0.09 (0.38)	-0.15 (0.30)	-0.12 (0.39)

On the first task, bias was less on the castle, *t*(78) = -2.21, *p* = 0.03, *d* = 0.50, with significant underestimation on the essay, *t*(39) = -4.30, *p* < 0.001, and non-significant underestimation on the castle, *t*(39) = -0.66, *p* = 0.516.

On the second task, a 2 (duration memory prompt) × 2 (task similarity) ANOVA produced no interaction, *F*(1,76) = 0.01, *MSE* = 0.01, *p* = 0.98, $\eta_{p}^{2}$ = 0.01, and no effect of duration memory prompt, *F*(1,76) = 0.22, *MSE* = 0.03, *p* = 0.641, $\eta_{p}^{2}$ = 0.01. There was an effect of task similarity, *F*(1,76) = 7.65, *MSE* = 0.90, *p* = 0.007, $\eta_{p}^{2}$ = 0.09, with prediction bias being less in the essay-first conditions (*M* = 0.08, *SD* = 0.33) than the castle-first conditions (*M* = -0.14, *SD* = 0.34). There was non-significant overestimation in the essay-first conditions, *t*(39) = 1.42, *p* = 0.164, and significant underestimation in the castle-first conditions, *t*(39) = -2.53, *p* = 0.016. Predictions on the second task were longer in essay-first condition (*M* = 34.80, *SD* = 10.05) than the castle-first condition (*M* = 31.23, *SD* = 7.66), and the difference approached significance, *t*(78) = 1.79, *p* = 0.08, *d* = 0.40. This finding suggests that the effect of task similarity was due mainly to the difference between the conditions in the actual duration of the second task (see above).

To examine the accuracy of memory for feedback previous task duration, the relationship between actual and recalled durations on the first task was assessed using Pearson correlations. Actual and recalled durations were highly correlated on both tasks overall, *r*(20) = 0.97, *p* < 0.001. High correlations were observed on the essay, *r*(20) = 0.95, *p* < 0.001, and the castle separately, *r*(20) = 0.98, *p* < 0.001, and the difference between these coefficients was not significant, Fisher *z* = 1.36, *p* = 0.174. These findings suggest that the feedback duration was accurately remembered.

To assess whether predictions were based on previous task duration, the relationship between predictions on the second task and the actual and recalled durations of the first task was examined using Pearson correlations. Predictions were quite weakly correlated with actual durations in the essay-first, *r*(20) = 0.20, *p* = 0.221, and castle-first memory prompt conditions, *r*(20) = 0.03, *p* = 0.834, and the coefficients did not differ significantly, Fisher *z* = 0.05, *p* = 0.617. Predictions and recalled durations were quite weakly correlated in the essay-first, *r*(20) = -0.11, *p* = 0.651, and castle-first memory prompt conditions, *r*(20) = -0.02, *p* = 0.946, and the coefficients did not differ significantly, Fisher *z* = -0.26, *p* = 0.795. These findings suggest that the accurately remembered task duration is not informative for predictions regardless of task similarity. In conjunction with the task similarity effect for prediction bias, these findings indicate that previous task duration feedback does not always serve to correct memory and debias predictions (Roy et al., 2008).

To assess whether anchoring predictions on previous task duration is influenced by task similarity, predictions on the second task were compared with the actual duration of the first task. Predicted and actual duration was similar in the castle-first memory-prompt (*M* = 29.15, *SD* = 8.27, and *M* = 32.40, *SD* = 7.59, respectively), *t*(19) = -1.43, *p* = 0.17, *d* = 0.39, and no memory-prompt conditions (*M* = 30.45, *SD* = 11.45, and *M* = 30.05, *SD* = 7.75, respectively), *t*(19) = 0.13, *p* = 0.90, *d* = 0.03. Predictions were shorter than actual durations in the essay-first no memory-prompt condition (*M* = 36.05, *SD* = 9.77, and *M* = 43.50, *SD* = 10.50, respectively), *t*(19) = 3.57, *p* = 0.002, *d* = 0.71, and the essay-first memory-prompt condition (*M* = 35.80, *SD* = 10.57, and *M* = 41.35, *SD* = 10.66, respectively), but the difference was not significant in the memory-prompt condition, *t*(19) = 1.54, *p* = 0.14, *d* = 0.52.

These findings suggest that predictions were anchored on previous task duration in both castle-first conditions, whereas anchoring was less clear in the essay-first conditions. Predictions being shorter than actual durations in the essay-first conditions could be a consequence of anchoring but in conjunction with the sufficient adjustment of predictions from the anchor according to the known demands of an identical second task. As the second essay tended to be completed faster than the first essay in the essay-first conditions, adjusting predictions away from an anchor of the duration of the same task would be expected to reduce prediction bias when the task was next performed.

Further likely support for the adjustment of predictions from the anchor of previous task duration comes from analysis of the difference between predictions on the second task and recalled durations on the first task in each memory-prompt condition. In the essay-first condition, predictions were shorter than recalled durations (*M* = 35.80, *SD* = 10.58, and *M* = 40.25, *SD* = 11.18, respectively), but not significantly so, *t*(19) = 1.23, *p* = 0.234, *d* = 0.28. In the castle-first condition, predictions were longer than recalled durations (*M* = 32.40, *SD* = 7.59, and *M* = 29.50, *SD* = 8.64, respectively), but not significantly so, *t*(19) = -1.12, *p* = 0.277, *d* = -0.26. These findings are indicative of some adjustment of predictions away from the anchor of quite accurately remembered previous task duration, but that this strategy was only useful for reducing bias when the identical essay task was performed for a second time.

To check whether the self-generated learning effect (König et al., 2015) occurred, error scores on the first and second tasks were compared between the task similarity conditions. A 2 (task) × 2 (task similarity) mixed ANOVA produced an interaction, *F*(1,78) = 14.16, *MSE* = 0.14, *p* < 0.001, $\eta_{p}^{2}$ = 0.15, with error scores being significantly higher on the first task than the second task in the essay-first condition (*p* < 0.001) but not significantly lower on the first task than the second task in the castle-first condition (*p* = 0.38). In conjunction with the task similarity effect, this finding suggests that participants that did the essay first learnt from their mistakes on that task and used this self-generated informative feedback to make a less biased prediction on the second task.

Consistent with Experiment 1, prediction bias was less on the second task when it was the same task as the first task. As duration feedback was provided, it seems that knowing how long the previous task took to complete reduces bias on the next task only when previous and focal tasks are identical. Moreover, the task similarity effect for bias occurred regardless of whether the recall of the feedback previous task duration was prompted just before making a prediction on the second task. As the two memory-prompt conditions recalled the duration of the first task quite accurately, the ineffectiveness of the memory prompt manipulation is rather surprising. Accurately recalling the feedback duration of the first essay task shortly before predicting the duration of the same task should have resulted in the least biased predictions. However, as duration feedback has been shown to reduce prediction bias when similar tasks are performed 1 week apart without any memory recall prompt (Roy et al., 2008), such a prompt might not be necessary because people’s memory for such feedback tends to be quite accurate. It could be that knowing the duration of the first task was sufficient to correct memory and reduce bias on the second task in the essay-first conditions, thus rendering the memory-prompt manipulation ineffective. Similarly, the feedback duration of the same previous task could have served to heighten learning from mistakes on that task (König et al., 2015) and this information was used to good effect when predicting the duration of the second task.

This experiment suggests that knowing the exact duration of a previous task does not necessarily correct memory and reduce prediction bias on the next task (Roy et al., 2008) regardless of the similarity of previous and focal tasks. Rather, it seems that task similarity reduces bias through learning from how the previous task was performed when making a prediction on the same task again (König et al., 2015). The task similarity effect might be due to the use of an anchoring strategy (Thomas and Handley, 2008) wherein predictions are sufficiently adjusted from the anchor of previous task duration according to the demands of the focal task only when the tasks are the same, meaning that the feedback duration is informative and relevant. However, as all participants were told how long the first task took them, the usefulness of duration feedback with respect to task similarity is speculative at best. Simply performing the essay task for a second time could have been sufficient to reduce prediction bias, thus negating any effect of duration feedback. It could be that participants in the essay-first conditions realized that they had misestimated on the first task and used this relevant information to make a less biased prediction on the second task.

To more fully address the role of previous task information, task duration feedback was manipulated in Experiment 3 alongside task similarity which was examined in the same way as in Experiments 1 and 2. Task duration feedback was manipulated by using control conditions in which an essay task was performed twice but feedback was not provided. If previous task duration feedback serves to reduce bias, predictions on the second task should be less biased in the feedback conditions, whereas if task similarity reduces bias, predictions should be less biased when the essay is performed twice regardless of whether duration feedback is provided. Additionally, the role of previous task information was further investigated in Experiment 3 by more directly assessing participants’ perceptions of the first task by having them report what information they used as a basis for their prediction on the second task, thus elucidating their reasoning processes. If duration feedback is beneficial when tasks are similar, then having this information could be reported as a major reason for predictions on the second essay task. Conversely, if task similarity is sufficient to reduce bias, then aspects of the first task could be reported as a major reason for predictions on the second essay task. The use of verbal protocols (Ericsson and Simon, 1980) to gauge the reasons participants gave for predictions on the second task is also beneficial in providing an insight into the kind of information people use when making time predictions on tasks that have or have not been performed recently (i.e., when prior task experience differs).

## Experiment 3

As in Experiments 1 and 2, all participants performed the same essay task at the second session and performed the same essay task or the castle task at the first session 1 week earlier. The duration of the first task was feedback to half of the castle-first and essay-first conditions at the end of the first session. Just after predicting the duration of the second task, all participants reported what they had based that prediction upon.

### Method

#### Participants

Eighty psychology students (24 male and 56 female) at a large university in Southern England participated voluntarily in return for course credit. Participants were aged 18 to 35 (*M* = 20.63, *SD* = 2.85) years.

#### Design

A 2 (Task Similarity [same vs. different]) × 2 (Previous Task Duration Feedback [yes vs. no]) between-groups design was used.

#### Materials

The castle task was from Experiment 2. The essay task was a shortened version of the essay task from Experiments 1 and 2. It was shortened to make it similar to the average duration of the castle task from Experiment 2 (29.80 min). Pilot-testing (*N* = 10) revealed that an essay comprising 1302 words and involving making 140 changes could be formatted in 30 min on average and this task was used.

#### Procedure

The procedure was identical to Experiment 2 except for the following three changes. One, at the start of the first session, participants were randomly assigned to one of four equal-sized conditions that were formed by crossing the task similarity and previous task duration feedback factors. Two, just after the first task had been completed, half of the essay-first and castle-first conditions were told exactly how much time they took to complete the first task. Three, just after predicting the duration of the second task, participants reported in writing what factors they had based their prediction upon.

### Results and Discussion

The data from Experiment 3 is available in **Supplementary Data Sheet S3**. At the first session, the essay condition made 124.40 corrections to the task on average (*SD* = 24.39) and all of the castle condition completed the task correctly. At the second session, the number of changes made to the essay did not differ between the essay-first (*M* = 126.60, *SD* = 5.24) and castle-first conditions (*M* = 128.68, *SD* = 6.39), *t*(78) = 1.59, *p* = 0.116, *d* = 0.33.

Basic descriptive statistics for the first and second tasks are presented in **Tables 4** and **5**, respectively. **Table 4** shows that there was underestimation on the castle and essay at the first session. The castle and essay tasks did not differ in duration, *t*(78) = 1.12, *p* = 0.267, *d* = 0.24. However, to be consistent with Experiments 1 and 2, proportional error scores were used to assess prediction bias on the first task. **Table 5** shows that there was overestimation in the essay-first conditions and underestimation in the castle-first conditions on the second task. At the second session, the castle-first conditions took longer to complete the essay (*M* = 31.38, *SD* = 8.09) than the essay-first conditions (*M* = 22.68, *SD* = 6.31), *t*(78) = -5.36, *p* < 0.001, *d* = 0.98. As the tasks differed in duration, proportional error scores were used to assess prediction bias on the second task.

**Table 4:** Mean (standard deviation) predicted and actual duration and prediction bias (minutes), and signed proportional error scores per the first task in Experiment 3.

	Task	
	Essay (*n* = 40)	Castle (*n* = 40)
Predicted duration	19.50 (7.83)	20.65 (9.71)
Actual duration	28.55 (8.39)	26.33 (9.39)
Prediction bias	-9.05 (7.68)	-5.68 (12.42)
Error score	-0.30 (0.25)	-0.16 (0.45)

**Table 5:** Mean (standard deviation) predicted and actual duration and prediction bias (minutes), and proportional error scores per task similarity by first task duration feedback condition on the second task in Experiment 3.

	Condition			
	Essay-first No Feedback	Essay-first Feedback	Castle-first No Feedback	Castle-first Feedback
	(*n* = 20)	(*n* = 20)	(*n* = 20)	(*n* = 20)
Predicted duration	25.00 (9.18)	22.15 (6.74)	23.10 (5.95)	25.45 (10.78)
Actual duration	24.10 (6.12)	21.25 (6.33)	32.15 (6.52)	30.60 (9.51)
Prediction bias	0.60 (6.72)	0.90 (3.58)	-9.05 (6.13)	-5.15 (7.12)
Error score	0.02 (0.28)	0.06 (0.17)	-0.27 (0.17)	-0.16 (0.21)

On the first task, a between-groups *t*-test revealed that prediction bias was less on the castle, *t*(78) = -1.70, *p* = 0.047, *d* = 0.39 (one-tailed). This finding suggests that the student participants were less good at predicting the duration of the more familiar essay-formatting task. One-sample *t*-tests with a test value of zero (no bias) revealed that the underestimation indicative of the planning fallacy (Kahneman and Tversky, 1979) was evident on the castle, *t*(39) = -2.22, *p* = 0.032, and the essay, *t*(39) = -7.60, *p* < 0.001.

A 2 (duration feedback) × 2 (task similarity) ANOVA on the second task produced no interaction, *F*(1,76) = 0.70, *MSE* = 0.11, *p* = 0.406, $\eta_{p}^{2}$ = 0.01, and no effect of duration feedback, *F*(1,76) = 2.52, *MSE* = 0.03, *p* = 0.116, $\eta_{p}^{2}$ = 0.03. There was an effect of task similarity, *F*(1,76) = 28.90, *MSE* = 1.29, *p* < 0.001, $\eta_{p}^{2}$ = 0.28, with predictions being less biased in the essay-first conditions (*M* = 0.04, *SD* = 0.23) than the castle-first conditions (*M* = -22, *SD* = 0.20). There was non-significant overestimation in the essay-first conditions, *t*(39) = 1.03, *p* = 0.311, and significant underestimation in the castle-first conditions, *t*(39) = -7.02, *p* < 0.001. Predictions on the second task were similar in the essay-first (*M* = 23.58, *SD* = 8.08) and castle-first conditions (*M* = 24.28, *SD* = 8.68), *t*(78) = -0.37, *p* = 0.710, *d* = 0.08, suggesting that the effect of task similarity was mainly due to the difference between the conditions in the actual duration of the second task (see above).

To ascertain whether predictions were based on previous task duration, predictions on the second task and actual durations on the first task were correlated per condition. Consistent with the memory-bias account, as duration feedback can correct memory (Roy et al., 2008), correlations should be greater in the feedback conditions than the no feedback conditions, and this effect might or might not be influenced by task similarity. Pearson correlations produced the following results. The correlation was strong in the essay-first feedback condition, *r*(20) = 0.87, *p* < 0.001, and moderately strong in the essay-first no feedback condition, *r*(20) = 0.68, *p* = 0.001, and the difference between these coefficients was not significant, Fisher *z* = 1.47*, p* = 0.143. The correlation was weak in the castle-first no feedback condition, *r*(20) = 0.04, *p* = 0.875, and moderately weak in the castle-first feedback condition, *r*(20) = 0.24, *p* = 0.302, and the difference between these coefficients was not significant, Fisher *z* = 0.60*, p* = 0.548 The correlation was strong in both essay conditions combined, *r*(20) = 0.75, *p* < 0.001 and weak in both castle conditions combined, *r*(20) = 0.16, *p* = 0.331, and the difference between the coefficients was significant, Fisher *z* = 2.37*, p* = 0.018. These findings suggest that memory for previous task duration is only informative for predictions on the next task when that task is the same task. In conjunction with the effect of task similarity, it seems that receiving feedback on previous task duration fails to serve to correct memory and debias predictions when it pertains to a different task (see Roy et al., 2008).

To check whether an anchoring prediction strategy was used, predictions on the second task were compared with actual durations on the first task per condition. Predicted and actual durations were similar in the castle-first feedback, *t*(19) = 0.52, *p* = 0.611, *d* = 0.17, and no feedback conditions, *t*(19) = 1.04, *p* = 0.312, *d* = 0.27, whereas predictions were shorter than actual durations in the essay-first feedback, *t*(19) = 3.72, *p* = 0.001, *d* = 0.43, and no feedback conditions, *t*(19) = 3.97, *p* = 0.001, *d* = 0.65. Consistent with the use of an anchoring strategy, these findings suggest that predictions were sufficiently adjusted for the demands of the second task only when a previous first task is informative for a focal task (Thomas and Handley, 2008).

Further support for the use of an anchoring strategy comes from the reasons that participants gave for their predictions on the second task. Three categories of reasons were identified by two independent raters: personal ability (e.g., using computers); the first task (e.g., duration); the second task (e.g., familiarity). The first task was cited as the reason for predictions on the second task by most participants in the castle-first, χ*^2^*(1, *N* = 40) = 19.60, *p* < 0.001, and essay-first conditions, χ*^2^*(2, *N* = 40) = 13.40, *p* = 0.001 (60 and 85%, respectively), with personal ability being cited by 15% of the essay-first conditions only. The number of participants that cited the first task did not differ between the castle-first and essay-first conditions, χ^2^(1, *N* = 58) = 1.72, *p* = 0.189. These findings suggest that predictions on the second task were based on elements of the first task. In conjunction with the task similarity effect, these findings indicate that people tend to based their predictions on what tasks they have done before and this strategy is only useful for reducing prediction bias when focal tasks are the same as previous tasks.

To check whether the self-generated learning effect (König et al., 2015) held when previous and focal tasks are identical, error scores on the first and second tasks were compared between the essay-first and castle-first conditions. A 2 (task) × 2 (task similarity) mixed ANOVA produced an interaction, *F*(1,78) = 19.97, *MSE* = 0.08, *p* < 0.001, $\eta_{p}^{2}$ = 0.20, with error scores being significantly higher on the first task than the second task in the essay-first condition (*p* < 0.001) but not significantly lower on the first task than the second task in the castle-first condition (*p* = 0.41). In conjunction with the task similarity effect, this finding suggests that participants learnt from their mistakes on the first task when it was the essay and used this self-generated informative feedback to make less biased predictions when faced with the same task again.

As in Experiments 1 and 2, predictions on the second task were less biased when the task was identical to the first task. This task similarity effect held regardless of whether or not participants were told the exact duration of the first task. The lack of an effect of duration feedback suggests that having such information does not always correct memory and debias predictions (Roy et al., 2008). It would seem that performing the same task a few days previously was sufficient to produce more realistic predictions, perhaps through learning from mistakes made on the first task and using this useful information to good effect. It is feasible that participants that received duration feedback recognized that their first prediction was incorrect and sought to rectify this error when making a second prediction. Using this kind of self-generated learning strategy (König et al., 2015) should result in less biased predictions on the same second task because of the relevance of the information about the previous task.

Consistent with the self-generated learning effect, most participants referred to the first task as the basis for their prediction on the second task regardless of the similarity of the two tasks. This finding suggests that previous tasks are considered when predicting task duration. Contrary to the inside–outside account (Kahneman and Lovallo, 1993), this finding implies that distributional information (Kahneman and Tversky, 1979) is used yet does not necessarily reduce prediction bias. Consistent with the anchoring (Thomas and Handley, 2008), memory-bias (Roy et al., 2005), and self-learning accounts (König et al., 2015), the finding suggests that information about previous tasks is used when misestimating upcoming task duration. Moreover, being told how long the same previous task took does not result in better predictions compared with not having this information, suggesting that the benefit of receiving duration feedback (Roy et al., 2008) is not universal.

## General Discussion

By examining task similarity and previous task duration feedback, this research provides a robust test of the effect of knowledge about previous tasks on bias in predictions of task duration. Moreover, studying task similarity permits a test of the four theoretical accounts of task duration prediction bias: inside–outside, memory-bias, anchoring, and self-learning. In all experiments, task similarity influenced bias as predictions were closer to the actual durations of the second task when it was the same essay as the first task. Also, task similarity seemed to prevail over being told the exact duration of the previous task beforehand when the recall of this feedback was manipulated (Experiment 2) and the provision of this feedback was manipulated (Experiment 3), as such duration feedback did not influence prediction bias.

In contrast to the work of Roy et al. (2008), the lack of any effect of previous task duration feedback suggests that having such information did not correct participants’ memory for the first task and debias their predictions on the second task. Rather, the task similarity effect is consistent with the notion that participants implicitly learnt from their mistakes on the first essay task and used this pertinent information to good effect when they made a prediction on the same task for a second time. Conversely, implicitly learning from mistakes made on the castle task would not be beneficial for reducing prediction bias because of the irrelevance of the self-generated feedback to the essay task (König et al., 2015). In all experiments, the difference in bias between the first and second tasks was greater when both tasks were the essay rather than the castle and the essay. Given the task similarity effect, this finding is consistent with the self-learning account but indicates that learning from one’s mistakes on a previous task does not serve to reduce prediction bias on the next task when that task is different from the previous task. Thus, this research highlights the role of task similarity in the self-learning account (König et al., 2015). Moreover, the present support for the self-learning account suggests that it has emerged as an important contributor to our understanding of the task duration prediction process with good explanatory power, thus constituting a valuable addition to the literature.

The task similarity effect for prediction bias also seems consistent with the anchoring account (e.g., Thomas and Handley, 2008), with predictions on the second task being based on the (feedback or perceived) duration of the first essay task but adjusted according to the demands of the second task. Such adjustment should be sufficient for the demands of the upcoming identical task because of the relevance of the information gleaned from performing the previous task, resulting in less bias. Conversely, adjusting predictions on the essay from the demands of the very different castle task is not informative of what performing the essay entails and so would not be expected to reduce bias.

There was not universal evidence for participants using such a strategy though, as predictions on the second task and actual durations on the first task differed only in both essay-first conditions in Experiment 2 and in the essay-first no memory-prompt condition in Experiment 3. The lack of a difference between these measures in Experiment 1, and in the essay-first memory-prompt condition in Experiment 2 and both castle-first conditions in Experiments 2 and 3 could be indicative of anchoring without adjustment. Consistent with this suggestion, Jacowitz and Kahneman (1995) found evidence of anchoring without adjustment on estimation tasks without a temporal element (e.g., estimating the distance between local cities) due to a process of comparing an anchor value with a target task value rather than providing an estimate of the target task value based on that same anchor value. Jacowitz and Kahneman (1995) created a two-stage judgment process to separate anchoring from adjustment, with the first stage being devoid of adjustment due to no estimation of the target task value being made, only a judgment comparing the target task value with the anchor value. It was found that comparison judgments were biased in the direction of the anchor value, with this process occurring before participants were required to make an estimate regarding the value of the target task. Thus, anchoring was evident when adjustment was not possible.

In the context of the present research, Jacowitz and Kahneman’s (1995) finding could have manifested itself in participants comparing the demands of the second task with the actual feedback duration of the first task (Experiments 1 and 2) and the perceived or actual feedback duration of the first task (Experiment 3) and using this information as an anchor for predictions on the second task. This strategy would be expected to yield greater prediction bias (significant underestimation) on the essay when the castle was performed first because the castle-first conditions took longer to do the essay than the essay-first conditions (in all experiments) due to a lack of prior task experience.

Although such an anchoring-only strategy could account for the effect of task similarity, the lack of a difference between the actual duration of the previous task and the predicted duration of the focal task when the tasks are identical and the exact duration of the previous task is known (Experiments 1 and 2) and remembered quite accurately (Experiment 2) is difficult to explain. As previous task durations and focal task predictions were similar in Experiment 1, it could be that using an anchoring-only strategy was effective in the essay-first conditions because those participants used the knowledge they gained from performing the first task along with the precise information they received about how long the task took to make a better prediction on the second task because on their task-specific experience. In the essay-first memory-prompt condition in Experiment 2, predictions on the second task were over 5 min shorter than actual durations on the first task on average (although not a reliable difference), suggesting that some downward adjustment might have occurred. In the essay-first no memory-prompt condition in Experiment 2, predictions on the second task were reliably shorter than actual durations on the first task, suggesting that some downward adjustment did occur.

The present findings are broadly consistent with the anchoring account (e.g., Thomas and Handley, 2008). However, incorporating separate manipulations of anchoring alone and anchoring and adjustment in future research would provide a more robust examination of the role of anchoring in task duration prediction bias as well as extending the work of Jacowitz and Kahneman (1995) to judgment tasks with a strong temporal element. Future research could examine anchoring in isolation by measuring the difference between anchor values of normative previous task durations presented to participants and participants’ own estimates of whether the same task would take them longer or shorter than the anchor values. In conjunction with the task similarity effect for prediction bias, such manipulations would further elucidate the cognitive mechanisms involved in predicting task duration.

Further support for the anchoring account derives from the difference in the direction of misestimation on the second task between the essay-first and castle-first conditions in all experiments. In Experiments 1 and 2, anchoring predictions on the feedback duration of the first task would yield underestimation when the shorter castle was performed first and slight (non-significant) overestimation when the same essay that took longer was performed first. Similarly, in Experiment 3, anchoring predictions on the perceived or feedback duration of the first task (no feedback and feedback conditions, respectively) would result in underestimation in the castle-first conditions because these participants tended to take longer to do the essay than participants in the essay-first conditions who also tended to do the second task quicker than the first task [*t*(39) = 6.18, *p* < 0.001, *d* = 0.70], resulting in slight (non-significant) overestimation. These findings suggest that the actual duration of a previous task can influence whether the underestimation indicative of the planning fallacy (Kahneman and Tversky, 1979) occurs on a focal task.

Although providing feedback on the actual duration of a previous task did not debias predictions on a focal task *per se*, there was some support for the memory-bias account (e.g., Roy et al., 2008) as predictions on the second task were more strongly correlated with actual durations on the first task in the essay-first conditions in Experiments 1 and 3. Coupled with the task similarity effect, this finding suggests that remembered previous task duration influences predictions on subsequent tasks but only serves to reduce bias when the tasks are the same.

Support for the memory-bias account was not universal, however, as predictions on the second task and actual durations on the first task were only weakly correlated in all conditions in Experiment 2. As the duration of the first task was recalled quite accurately in the castle-first and essay-first memory-prompt conditions in Experiment 2, the lack of a strong correlation in the essay-first conditions is not easily explicable, especially as recalled and actual durations on the first task were similar in both memory-prompt conditions. Given the manipulation of previous task duration feedback in Experiment 3 that experiment seems to provide a better barometer for the memory-bias account. The stronger correlation between previous task durations and focal task predictions in the essay-first feedback condition than the castle-first feedback condition in Experiment 3 indicates that task duration feedback does not serve to correct memory and debias predictions when previous and focal tasks are different. Thus, it seems that the memory-bias account should be revised to incorporate task similarity, especially with respect to the role of duration feedback.

Consistent with the memory-bias, self-learning, and anchoring accounts, this research suggests that information about previous tasks is considered when predicting task duration, but does necessarily reduce bias as the inside–outside account implies (e.g., Buehler et al., 1994). Moreover, in accordance with Roy et al.’s (2008) research, Experiment 2 indicates that the feedback duration of a previous task is recalled quite accurately 1 week after being provided. It could be that the quite lengthy interval between the first and second tasks in the present research was not sufficient to eliminate the memory-trace of the feedback duration of the first task because this information was provided just after the first task was performed. Thus, the immediacy of the feedback provided in Experiment 2 might have consolidated participants’ memory for the duration of the first task and facilitated learning for participants that performed the essay task twice, resulting in less prediction bias on the second essay task.

This research highlights the effect of similarity on task duration prediction bias. It has been shown that predictions are less biased when the same task is performed 1 week previously. In addition, by providing accurate feedback on the duration of a just-completed identical or different task (Experiments 1 and 2) and manipulating the provision of such information (Experiment 3), the research demonstrates that having such knowledge is an effective method for reducing misestimation only when a previous task is the same as a focal task. The research provides consistent support for the self-learning account and as well as some support for the anchoring and memory-bias accounts, and suggests that information about previous task performance is used when predicting task duration. However, contrary to the inside–outside account, the research suggests that using such information does not necessarily reduce prediction bias. Importantly, the research calls into question the claim that ignoring information about previous tasks can explain the planning fallacy and task duration prediction bias (e.g., Buehler et al., 1994).

The present research indicates that the similarity of previous and focal tasks is an important factor in the process of predicting task duration effectively. The basis of the effect of task similarity was mainly due to differences between the essay-first and castle-first conditions in actual durations on the second task, with predictions on the essay only differing between these conditions in Experiment 1. In all experiments, the essay task in the second session was performed in less time when the same task was performed in the first session compared with when the very different castle task was performed in the first session. Coupled with the task similarity effect, this finding suggests that the similarity of a previous task can influence actual behaviors, with performance on a focal task being facilitated when that same task has been completed 1 week before. The positive effect of task similarity on actual behavior provides further support for the self-learning account (König et al., 2015) by indicating that the insight gained from knowing about one’s mistakes on a previous similar task can be transferred to not only predicting the duration of the next task better but also performing that task quicker.

The reasons that participants in Experiment 3 gave for their prediction on the second task are supportive of the finding that previous task similarity can influence actual behaviors on the next task as the first task was referred to by most participants in the essay-first and castle-first conditions. This finding suggests that the task that people have done most recently in a given environment is what they base their prediction on regarding how long they believe the next task in that same environment will take to perform. Basing predictions on how well one performed on a previous task should not reduce bias on the next task when that task is very different because the characteristics of the tasks are heterogeneous (Thomas et al., 2007). As retrospective verbal reports might not the most robust form of data (Ericsson and Simon, 1980), future research should create experimental manipulations to unpack the effect of task similarity on actual behaviors as well as predictions of task duration. Doing so would elucidate the cognitive mechanisms underpinning time prediction as well as the transfer of insight between one’s performance on previous and focal tasks, thus further testing the self-learning account (König et al., 2015).

Although the present research makes an important contribution to the literature by enhancing our understanding the time prediction process, it also gives rise to some ideas for developing the field further. One avenue that could potentially bolster support for the self-learning account is to examine whether the transfer of insight from previous to focal tasks results in reduced prediction bias when the focal task is an unfamiliar one with which participants have limited or no prior experience. The present focus on the effect of task similarity with a familiar type of focal task precluded the use of a fully crossed factorial experimental design, which did not compromise the interpretation of the findings but meant that the role of task similarity was not explored as fully as it could have been. By manipulating the familiarity of focal and previous tasks along with prior task experience, future research will be able to delineate the time prediction process more fully, thus permitting a further test of the self-learning account as well as the other theoretical accounts.

Another seemingly fruitful direction for future research to further test all four accounts would be to examine the role of individual differences in feeling states and task engagement as it has been shown that these factors can differentially influence performance on tasks requiring different mental operations (Matthews et al., 2002; see also Langner et al., 2010). For example, using scores on the short version of the Dundee Stress State Questionnaire (DSSQ; Matthews et al., 2002), which assesses subjective experience in task performance situations, Matthews et al. (2002) found higher distress, lower worry and some increase in task engagement was evident for tasks involving working memory (e.g., mental arithmetic), whereas for tasks involving vigilance (e.g., watching images), which are less mentally demanding, lower worry, lower task engagement, and some distress was evident. Such research has potentially important implications for the field of task duration prediction as support for the planning fallacy (Kahneman and Tversky, 1979) and temporal misestimation in general has been observed on a diverse range of tasks (see Buehler et al., 2010a, for a review), which are highly unlikely to be uniform in terms of the mental demands they place on the people performing them. Thus, examining the interplay between task type and individual differences in task engagement and stress state would provide a welcome addition to the task duration prediction literature. In the context of the present research, it could be that the essay-formatting task requires the kind of mental demands that apply to tasks involving vigilance (e.g., searching for grammatical errors) and so could be characterized by lower task engagement. Through using established psychometric measures like the DSSQ and systematically varying the type of tasks that people perform, future research will be able to provide an even more thorough account of the task duration prediction process and its determinants.

A final noteworthy avenue for future research concerns evaluating the way in which prediction bias is measured to ascertain whether proportional error scores are the most appropriate measure of bias when it comes to repeated exposure to the same task or similar tasks. Specifically, to determine whether there are test–retest effects at play that reduces the reliability of error scores as a suitable dependent measure. For example, being exposed to the present essay-formatting task for the second time in 7 days might mean that participant in the essay-first conditions automatically incorporated what they had learnt from performing the task the first time around into their prediction on the second trial of the task, thus influencing the error score derived from their performance on the second task.

Test–retest effects in relation to the speed and accuracy of task performance were examined by Steinborn et al. (2018) who took a psychometric approach and used a letter-checking task designed to assess sustained attention. The research revealed that the speed of repeated performance on the task was more reliable a measure of task performance accuracy relative scores based on errors, suggesting that such scores are not suitable as a principal measure of task performance accuracy. Although proportional error scores have been used widely in the task duration prediction literature (e.g., Roy et al., 2008; Thomas and Handley, 2008), and are an established measure of temporal misestimation (Brown, 1997), which takes account of differences in the speed of task performance (i.e., actual durations), their susceptibility to the effects of repeated task exposure has not been examined. Whilst assessing the robustness of error scores is beyond the scope of the present research, the worth of doing so in future research should be recognized if we are to gain a sufficiently thorough picture of the task duration prediction process.

By examining two key factors that influence the task duration prediction process in tandem, this research has arguably raised more questions than it has answered. For example, does the effect of task similarity occur when the interval between previous and focal tasks is longer or shorter than 1 week? Does using the duration of a previous similar or different task as an anchor for predictions influence bias only when there is scope to adjust predictions from anchor values according to the demands of focal tasks? Can variations in predictions on focal tasks as well as in actual durations of previous tasks explain the effect of task similarity when previous task duration feedback is not available? By answering such questions, future research will be even better-placed to identify the gamut of factors that are germane to the process of predicting task duration.

## Ethics Statement

The research presented in the manuscript was conducted in accordance with the ethical principles of the American Psychological Association concerning research with human participants. The protocol for the research was approved by Bournemouth University’s Science, Technology and Health Research Ethics Panel. All participants involved in the research gave their written informed consent in accordance with the Declaration of Helsinki.

## Author Contributions

KT and CK designed the experiments, gathered the experimental stimuli equally, and contributed equally to analyzing the data and writing the manuscript. KT oversaw the day–day running of the research (data collection, etc.).

## Conflict of Interest Statement

The authors declare that the research was conducted in the absence of any commercial or financial relationships that could be construed as a potential conflict of interest.

## Supplementary Material

DATA SHEET S1 Raw data from Experiment 1.

DATA SHEET S2 Raw data from Experiment 2.

DATA SHEET S3 Raw data from Experiment 3.

## References

[B1] Boltz M. G. Kupperman C. Dunne J. (1998). The role of learning in remembered duration. 26 903–921. 10.3758/BF03201172 9796225

[B2] Brown S. W. (1997). Attentional resources in timing: interference effects in concurrent temporal and nontemporal working memory tasks. 59 1118–1140. 10.3758/BF03205526 9360484

[B3] Buehler R. Griffin D. MacDonald H. (1997). The role of motivated reasoning in optimistic time predictions. 23 238–247. 10.1177/0146167297233003

[B4] Buehler R. Griffin D. Peetz J. (2010a). “The planning fallacy: cognitive, motivational, and social origins,” in Vol. 43 ed. Zanna M. (Burlington: Academic Press), 1–62. 10.1016/S0065-2601(10)43001-4

[B5] Buehler R. Griffin D. Ross M. (1994). Exploring the “planning fallacy”: why people underestimate their task completion times. 67 366–381. 10.1037/0022-3514.67.3.366

[B6] Buehler R. Peetz J. Griffin D. (2010b). Finishing on time: when do predictions influence completion times? 11 23–32. 10.1016/j.obhdp.2009.08.001

[B7] Burt C. D. B. (1992). Reconstruction of the duration of autobiographical events. 20 124–132. 10.3758/bf03197160 1565010

[B8] Burt C. D. B. Kemp S. (1991). Retrospective duration estimation of public events. 19 252–262. 10.3758/bf03211149 1861611

[B9] Epley N. Gilovich T. (2005). When effortful thinking influences judgmental anchoring: differential effects of forewarning and incentives on self-generated and externally provided anchors. 18 199–212. 10.1002/bdm.495

[B10] Ericsson K. A. Simon H. A. (1980). Verbal reports as data. 87 215–251. 10.1037/0033-295x.87.3.215

[B11] Francis-Smythe J. A. Robertson I. T. (1999). On the relationship between time management and time estimation. 90 333–347. 10.1348/000712699161459

[B12] Hinds P. (1999). The curse of expertise and debiasing methods on predictions of novice performance. 5 205–221. 10.1037/1076-898X.5.2.205

[B13] Jacowitz K. E. Kahneman D. (1995). Measures of anchoring in estimation tasks. 21 1161–1166. 10.1177/01461672952111004 2302814

[B14] Kahneman D. Lovallo D. (1993). Timid choices and bold forecasts: a cognitive perspective on risk taking. 39 17–31. 10.1287/mnsc.39.1.17

[B15] Kahneman D. Tversky A. (1979). Intuitive prediction: biases and corrective procedures. 12 313–327.

[B16] König C. J. (2005). Anchors distort estimates of expected duration. 96 253–256. 10.2466/PR0.96.2.253-256 15941095

[B17] König C. J. Wirz A. Thomas K. E. Weidmann R. Z. (2015). The effects of previous misestimation of task duration on estimating future task duration. 34 1–13. 10.1007/s12144-014-9236-3

[B18] Kruger J. Evans M. (2004). If you don’t want to be late, enumerate: unpacking reduces the planning fallacy. 40 586–598. 10.1016/j.jesp.2003.11.001

[B19] Langner R. Steinborn M. B. Chatterjee A. Sturn W. Willmes K. (2010). Mental fatigue and temporal preparation in simple reaction-time performance. 133 64–72. 10.1016/j.actpsy.2009.10.001 19878913

[B20] Matthews G. Campbell S. E. Falconer S. Joyner L. A. Huggins J. Gilliland K. (2002). Fundamental dimensions of subjective state in performance settings: task engagement, distress, and worry. 2 315–340. 10.1037//1528-3542.2.3.315 12899368

[B21] Roy M. M. Christenfeld N. J. S. (2007). Bias in memory predicts bias in estimation of future task duration. 35 557–564. 10.3758/bf03193294 17691153

[B22] Roy M. M. Christenfeld N. J. S. McKenzie C. R. M. (2005). Underestimating the duration of future events: memory incorrectly used or memory bias? 131 738–756. 10.1037/0033-2909.131.5.738 16187856

[B23] Roy M. M. Mitten S. T. Christenfeld N. J. S. (2008). Correcting memory improves accuracy of predicted task duration. 14 266–274. 10.1037/1076-898X.14.3.266 18808280

[B24] Steinborn M. B. Langner R. Flehmig H. C. Huestegge L. (2018). Methodology of performance scoring in the d2 sustained-attention test: cumulative-reliability functions and practical guidelines. 30 339–357. 10.1037/pas0000482 28406669

[B25] Thomas K. E. Handley S. J. (2008). Anchoring in time estimation. 127 24–29. 10.1016/j.actpsy.2006.12.004 17276378

[B26] Thomas K. E. Handley S. J. Newstead S. E. (2007). The role of prior task experience in temporal misestimation. 60 230–240. 10.1080/17470210600785091 17455056

[B27] Thomas K. E. Newstead S. E. Handley S. J. (2003). Exploring the time prediction process: the effects of task experience and complexity on prediction accuracy. 17 655–673. 10.1002/acp.893

[B28] Tversky A. Kahneman D. (1974). Judgment under uncertainty: heuristics and biases. 185 1124–1131. 10.1126/science.185.4157.1124 17835457

